# From Ion to Iron: A Focused Narrative Literature Review of Magnesium Supplementation for Muscle Strength and Recovery

**DOI:** 10.7759/cureus.115503

**Published:** 2026-08-31

**Authors:** Valerie Domingo, Cason Chaffee, Francisco Ibarra

**Affiliations:** 1 Department of Biomedical Education, College of Osteopathic Medicine, California Health Sciences University, Clovis, USA

**Keywords:** body composition, dietary supplements, exercise performance, inflammation, magnesium, muscle recovery, sports nutrition

## Abstract

Magnesium is an essential mineral involved in numerous physiological processes, including energy metabolism, neuromuscular function, protein synthesis, and inflammatory regulation. Given these roles, magnesium supplementation has received increasing attention as a potential strategy to support exercise performance and recovery. This review aimed to evaluate the current evidence regarding magnesium intake and its effects on exercise performance, skeletal muscle recovery, body composition, and inflammatory responses. A focused narrative literature review was conducted using PubMed to identify studies published between 2000 and 2025 that contained the terms “magnesium” and “muscle” in the title. Review articles, meta-analyses, animal studies, commentaries, guidelines, and studies lacking clinical muscle-related outcomes were excluded. Outcomes of interest included exercise performance, recovery measures, body composition, and inflammatory markers. Of 108 records identified, 10 studies met inclusion criteria, including six randomized controlled trials and four observational studies. Evidence regarding exercise performance was inconsistent. Some randomized trials demonstrated improvements in muscular strength, functional mobility, perceived exertion, and repetitions to failure, whereas others reported no performance benefit or reductions in maximal oxygen uptake (VO₂max) and sprint power with higher-dose supplementation. Recovery-related outcomes were generally more favorable, with several studies reporting reduced muscle soreness, improved perceived recovery, and lower markers of exercise-induced muscle damage. Magnesium intake was also associated with lower concentrations of inflammatory biomarkers, including high-sensitivity C-reactive protein, tumor necrosis factor-α, and interleukin-6. Observational studies consistently demonstrated associations between higher dietary magnesium intake and greater skeletal muscle mass, improved body composition, and lower systemic inflammation. Current evidence suggests that magnesium supplementation may provide greater benefit for exercise recovery and inflammatory regulation than for direct enhancement of athletic performance. Variability in study design, magnesium formulation, supplementation dose, participant characteristics, and baseline magnesium status likely contributed to the inconsistent performance findings observed across studies. Magnesium supplementation may be most appropriately considered an adjunct recovery-support strategy rather than a primary ergogenic aid. Large, well-designed randomized controlled trials are needed to clarify optimal dosing strategies, identify populations most likely to benefit, and further elucidate the mechanisms through which magnesium influences muscle function and exercise adaptation.

## Introduction and background

According to the CDC, approximately 58% of U.S. adults aged 20 years and older used a dietary supplement in the previous 30 days [1]. Sports supplements are among the most commonly used dietary products among athletes and physically active individuals seeking to support health, enhance recovery, and improve performance. Common examples include protein supplements, creatine, branched-chain amino acids, caffeine, and dehydroepiandrosterone (DHEA), which are used to support muscle recovery, energy production, endurance, and strength enhancement [2].

Magnesium has emerged as a supplement of growing interest in sports nutrition due to its essential role in cellular metabolism and neuromuscular function. Obtained through both dietary sources and supplemental formulations, magnesium acts as a cofactor in more than 300 enzymatic reactions involved in adenosine triphosphate (ATP) production, protein synthesis, and ion transport [3]. Beyond its established clinical utility in conditions such as hypomagnesemia, arrhythmias, migraines, and muscle cramps, magnesium may also influence exercise performance and recovery [4]. Several biologically plausible mechanisms have been proposed to explain these potential effects. Magnesium is required for ATP-dependent reactions involved in cellular energy production and may therefore influence exercise capacity and muscular function. As a physiologic calcium antagonist, magnesium helps regulate neuromuscular excitability, muscle contraction, and muscle relaxation [5]. Magnesium may also modulate inflammatory and oxidative stress pathways that influence post-exercise recovery, including muscle soreness, perceived recovery, exercise-induced muscle damage, and inflammatory biomarkers such as high-sensitivity C-reactive protein (hs-CRP) [6]. Furthermore, magnesium deficiency has been associated with impaired muscle function, fatigue, and increased inflammatory responses, providing a rationale for supplementation in individuals with inadequate intake or suboptimal magnesium status. However, the extent to which these proposed mechanisms translate into meaningful improvements in performance or recovery, and whether supplementation provides similar benefits in individuals with adequate magnesium intake, remain incompletely understood.

Objectives

This focused narrative literature review aimed to evaluate the available evidence regarding magnesium intake and its effects on exercise performance, skeletal muscle recovery, body composition, and inflammatory outcomes to better inform evidence-based supplementation practices.

Methodology

A focused narrative literature review was conducted using PubMed, selected for its extensive coverage of biomedical and clinical research literature. Articles published between 2000 and 2025 containing the terms “magnesium” and “muscle” in the title were screened. This search strategy was intentionally designed to identify studies with a primary focus on magnesium and muscle-related outcomes rather than to serve as a comprehensive systematic review. Review articles, animal studies, meta-analyses, commentaries, guidelines, studies without clinical muscle outcome data, studies without muscle-performance measures, or data unrelated to muscle strength or recovery were excluded. The endpoints evaluated included recovery outcomes, exercise performance, body composition, and inflammatory markers.

## Review

The search strategy is shown in Figure 1. The search identified 108 articles, of which 10 met inclusion criteria [7-16]. These included six randomized controlled trials and four observational studies evaluating the effects of magnesium intake on exercise performance, recovery outcomes, inflammatory markers, and body composition. The intervention studies examined a variety of magnesium formulations, including glycinate, oxide, and chloride salts, with supplementation doses ranging from 250 to 600 mg/day and intervention durations between seven days and eight weeks. Observational studies primarily evaluated dietary magnesium intake and its associations with muscle mass, body composition, and systemic inflammation. A summary of study characteristics and primary findings is presented in Table 1.

**Figure 1:**
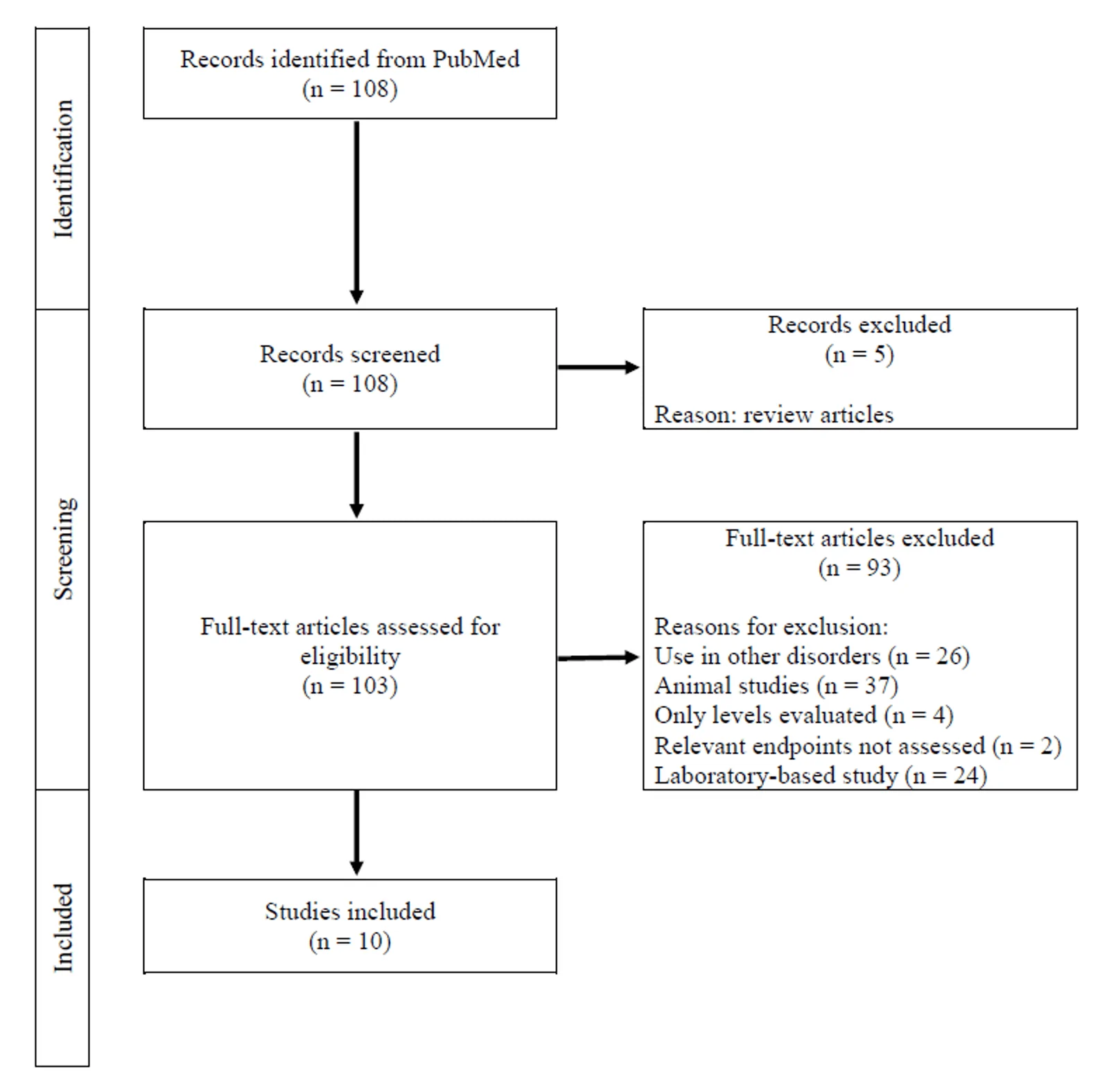
Literature search strategy.

**Table 1:** Summary of included studies. VO₂max: maximal oxygen uptake; RTF: total repetitions to failure; IU: international units

Studies	Study design	Sample size	Intervention	Key findings
Reno et al. (2022) [7]	Randomized controlled trial (RCT)	22 college-aged adults	Magnesium glycinate 350 mg/day for 10 days vs. placebo	Performance outcomes (total repetitions to failure, 65% RTF, 75% RTF) approached statistical significance in favor of magnesium; perceived recovery significantly improved in the magnesium group; muscle soreness significantly decreased from baseline in the magnesium group; ratings of perceived exertion decreased with magnesium but increased with placebo
Moslehi et al. (2013) [8]	RCT	69 middle-aged overweight women	Magnesium oxide 250 mg/day for 8 weeks vs. placebo	Handgrip strength improved from baseline in both groups; Timed Get Up and Go (TGUG) improved only in the magnesium group; body fat percentage decreased, and lean body mass increased within the magnesium group; no significant between-group differences in performance or body composition outcomes
Kheyruri et al. (2021) [9]	RCT	83 middle-aged adults	Magnesium oxide 250 mg/day plus vitamin D 50,000 IU weekly for 8 weeks vs. placebo	Significant improvements in upper- and lower-extremity strength and TGUG performance in the magnesium group; between-group improvements favored magnesium for handgrip strength and TGUG time; no significant body composition changes within and between groups; significant reductions in high-sensitivity C-reactive protein (hs-CRP) and tumor necrosis factor (TNF)-α within the magnesium group; only hs-CRP significantly lower vs. placebo
Steward et al. (2019) [10]	Crossover RCT	Nine adults in their late 20s	Magnesium supplementation 500 mg/day (magnesium oxide, magnesium stearate, and microcrystalline cellulose) for 7 days with 2-week washout vs. placebo	No significant improvement in 10-km downhill treadmill performance; interleukin (IL)-6 levels significantly reduced with magnesium; soluble IL-6 receptor levels significantly increased with magnesium; perceived muscle soreness significantly lower following magnesium supplementation
Córdova et al. (2019) [11]	RCT	18 adults in a 3-week cycling stage race	Magnesium supplementation (salt form not specified) 400 mg/day for 3 weeks vs. placebo	Myoglobin levels were significantly lower in the magnesium group during the race; no differences in creatinine, creatine kinase, lactate dehydrogenase, or cortisol; no significant differences in perceived exertion between groups
Bomar et al. (2025) [12]	Crossover RCT	15 adults in their 20s	Magnesium chloride 600 mg/day for 9 days with 3-week washout vs. placebo	VO₂max significantly decreased following magnesium supplementation; sprint mean power output significantly decreased following magnesium supplementation; no significant effect on 10-km time trial performance
Dong et al. (2022) [13]	Observational study	766 adolescents (14-18 years)	Dietary magnesium intake assessed by dietary recall (mean ≈200 mg/day)	Lower magnesium intake associated with higher hs-CRP levels; lower magnesium intake associated with lower fat-free mass; low magnesium amplified the negative relationship between inflammation and muscle mass
Welch et al. (2016) [14]	Cross-sectional study	2,570 middle-aged women	Dietary magnesium intake assessed by dietary recall	Higher magnesium intake associated with greater skeletal muscle mass; higher magnesium intake associated with greater leg explosive power; higher hs-CRP associated with lower muscle mass; higher magnesium intake attenuated the negative relationship between inflammation and muscle mass
Wang et al. (2024) [15]	Cross-sectional study	10,279 U.S. adults with hypertension	Dietary magnesium intake assessed by dietary recall	Higher magnesium intake associated with greater muscle mass; every 100 mg/day increase in magnesium associated with a 0.04 kg/m² increase in muscle mass index
Zhu et al. (2025) [16]	Cross-sectional study	5,793 adults (20-59 years)	Dietary magnesium intake assessed by dietary recall	Higher magnesium intake associated with higher skeletal muscle index; higher magnesium intake associated with lower risk of low muscle mass; highest intake group had significantly lower odds of low muscle mass compared with the lowest intake group

Exercise performance

Evidence regarding exercise performance was inconsistent. Several randomized trials demonstrated improvements in muscular strength, functional mobility, perceived exertion, or repetitions to failure following magnesium supplementation [7-9]. However, other studies reported no significant effects on endurance performance or exercise capacity, and one trial found reductions in maximal oxygen uptake (VO₂max) and sprint power following short-term high-dose supplementation [10-12]. Collectively, the available evidence does not support a uniform ergogenic effect of magnesium across all forms of exercise.

Recovery outcomes

Recovery-related outcomes demonstrated greater consistency. Multiple studies reported reductions in perceived muscle soreness and improvements in subjective recovery measures following supplementation [7,10]. Some investigations also observed favorable effects on markers of exercise-induced muscle damage, suggesting a potential role for magnesium in supporting post-exercise recovery [11].

Inflammatory markers

Several studies evaluated inflammatory outcomes and generally reported favorable findings. Magnesium supplementation was associated with reductions in biomarkers such as high-sensitivity C-reactive protein (hs-CRP), tumor necrosis factor (TNF)-α, and IL-6, while observational studies demonstrated inverse associations between dietary magnesium intake and systemic inflammation [9,10,13,14]. These findings suggest a potential anti-inflammatory effect that may contribute to recovery-related benefits.

Body composition

Observational studies consistently demonstrated associations between higher magnesium intake and greater skeletal muscle mass, improved fat-free mass, and lower risk of low muscle mass [13-16]. In contrast, supplementation trials generally failed to show significant improvements in body composition measures, suggesting that long-term dietary magnesium exposure may influence body composition more than short-term supplementation interventions [8,9].

Discussion

This review evaluated the current evidence regarding magnesium intake and its effects on exercise performance, recovery, inflammation, and body composition. Overall, the findings suggest that magnesium supplementation may be more beneficial for recovery-related outcomes and inflammatory regulation than for direct enhancement of athletic performance. However, considerable heterogeneity among studies makes definitive conclusions challenging.

The effects of magnesium supplementation on exercise performance varied substantially across studies. Several factors may account for these discrepancies. First, magnesium formulations differed considerably among investigations, with studies utilizing magnesium glycinate, oxide, chloride, and unspecified preparations [7-12]. These formulations differ in bioavailability and gastrointestinal absorption, potentially influencing biological effects and clinical outcomes [17]. Additionally, supplementation doses ranged from 250 to 600 mg/day, making direct comparisons difficult and raising the possibility that higher doses may not necessarily confer greater benefits. Differences in participant characteristics may have also contributed to variability in outcomes. Included studies enrolled diverse populations ranging from sedentary or overweight middle-aged adults to recreationally active and endurance-trained individuals. The effects of magnesium supplementation may be more pronounced in individuals with inadequate dietary intake than in those with sufficient baseline magnesium stores, but this hypothesis has not been adequately evaluated and warrants further investigation [6]. Notably, baseline magnesium concentrations were rarely assessed, limiting interpretation of whether observed benefits resulted from correction of deficiency or supplementation beyond physiological requirements. Variation in outcome measures further complicates interpretation. Some studies evaluated muscular endurance, others assessed functional mobility, maximal oxygen consumption, sprint power, or subjective ratings of exertion [7-9,11,12]. These measures likely reflect distinct physiological processes and may not respond similarly to magnesium supplementation. Consequently, inconsistent findings across performance domains should not be interpreted as contradictory, but rather as evidence that magnesium's effects may be context-specific.

Compared with performance outcomes, the evidence supporting benefits for recovery and inflammation was more consistent. Multiple studies reported reductions in muscle soreness, improvements in perceived recovery, and favorable changes in inflammatory biomarkers following supplementation [7,9,10]. These findings suggest that magnesium may exert its greatest effects through modulation of physiological stress responses rather than through direct enhancement of exercise capacity. Several biologically plausible mechanisms have been proposed to explain these findings. Magnesium is involved in energy metabolism, neuromuscular function, cellular signaling, and inflammatory regulation, all of which may influence the recovery process [4-6]. However, these mechanisms remain hypotheses rather than definitively established pathways, and further investigations are needed to clarify how magnesium affects post-exercise adaptation. The interpretation of recovery outcomes should also consider the subjective nature of many endpoints. Measures such as perceived soreness, recovery, and exertion are clinically relevant and meaningful to athletes, but they remain susceptible to expectation effects and reporting bias. Future studies should include both subjective and objective recovery markers to better characterize the effects of supplementation.

An important observation in this review was the discrepancy between observational and interventional evidence regarding body composition. Across multiple observational studies, higher dietary magnesium intake was consistently associated with greater skeletal muscle mass and more favorable body composition profiles [13-16]. However, these relationships were not consistently reproduced in the randomized controlled trials evaluating supplementation [8,9]. Several explanations may account for this difference. Observational studies may capture the cumulative effects of long-term dietary habits, whereas supplementation trials were generally short in duration and may have been insufficient to produce measurable changes in muscle mass. Additionally, individuals with higher magnesium intake may engage in healthier lifestyle behaviors, consume higher-quality diets, or participate in greater levels of physical activity, all of which may influence body composition independently of magnesium itself [18]. Therefore, while magnesium intake appears associated with favorable body composition outcomes, causality remains uncertain.

Limitations

Several limitations must be acknowledged. Many included randomized trials were characterized by small sample sizes and short intervention periods, limiting statistical power and generalizability. Heterogeneity in participant populations, magnesium formulations, dosing regimens, and outcome measures further complicates comparison across studies. Many recovery outcomes relied on subjective assessments, increasing susceptibility to reporting bias. Baseline magnesium status was infrequently measured, limiting interpretation of treatment effects. Additionally, the search strategy was limited to PubMed and title-based search terms. Although this focused approach identified studies with direct relevance to magnesium and muscle-related outcomes, potentially relevant studies indexed in other databases or identified through broader search strategies may have been missed. Therefore, the findings should be interpreted as a focused synthesis of the literature rather than an exhaustive systematic review.

## Conclusions

Current evidence suggests that magnesium may play a supportive role in skeletal muscle health, exercise recovery, and inflammatory regulation, although its effects on athletic performance remain inconsistent. Randomized controlled trials have demonstrated potential benefits for reducing muscle soreness, improving perceived recovery, and attenuating selected inflammatory responses to exercise. In contrast, evidence supporting improvements in strength, endurance performance, or body composition is less consistent and appears to be influenced by factors such as magnesium formulation, supplementation dose, participant characteristics, and baseline magnesium status. Observational studies consistently associate higher dietary magnesium intake with greater muscle mass and more favorable body composition, but these findings have not been reliably replicated in supplementation trials. Overall, magnesium supplementation may be most appropriately considered an adjunct recovery support strategy rather than a primary ergogenic aid. Additional well-designed randomized controlled trials with standardized outcome measures, assessment of baseline magnesium status, and longer intervention durations are needed to clarify optimal dosing strategies, identify populations most likely to benefit, and further elucidate the mechanisms underlying magnesium's effects on muscle function and exercise adaptation.
